# Abstracts

**DOI:** 10.1155/S146392468400002X

**Published:** 1984


Journal of Automatic Chemistry, Volume 6, Number 1 (January–March 1984), pages 3–5

### Abstracts

### Page 6

### Automation in analytical chemistry—from rule of thumb to fully automated methods. Some philosophies and social consequences

R. Stanley

This contribution sets out to show how techniques in analytical chemistry evolved from guesswork, conjecture and sometimes from empirical findings into a definitive subject now called analytical science, which embraces many technologies, strategies and philosophies not formerly encompassed in the more classical rigours of the practice of analytical chemistry. Though the history of analytical chemistry is complex and detailed and involves the lifeworks of many eminent scientists, there appears to exist quite definite landmarks which have dictated the ways in which analytical procedures have been carried out—particularly in the very recent past. Such ways have opened up a vast arena of social and economic problems and benefits which the world of microcomputers sets out to solve or justify. As such analytical strategies have become interdisciplinary and multi-dimensional, Britain appears to have failed to fully exploit the instrument business in modern times, and some reasons are given.

### L'automation dans la chimie analytique, de la simple règle à la méthode complètement automatisée. Aspects philosophiques et conséquences sociales

R. Stanley

Cet essai montre comment les techniques en chimie analytique ont évolué de la devinette ou parfois de la trouvaille empirique en un sujet bien défini aujourd'hui appelé la science analytique qui comprend des technologies, stratégies et philosophies autrefois excluses de la pratique classique de la chimie analytique. Bien que l'histoire de la chimie analytique soit complexe et qu'elle inclut l'oeuvre de tant de scientifiques éminents, il apparaît qu'ils existent des signes bien définis qui ont dicté les chemins dans lesquels les procédures analytiques ont été effectuées—particulièrement lors les dernières années. Ces chemins ont ouvert un grand nombre de problèmes sociaux et économiques et des bénéfices qui doivent être résolus ou justifiés par le monde des micro-ordinateurs. Telles que les stratégies analytiques sont devenues interdisciplinaires et multi-dimensionnelles. La Grande-Bretagne apparaît avoir raté la chance d'exploiter le marché instrumental des temps modernes et quelques raisons sont données.

### Automation in der analytischen Chemie von Daumenregel—bis zu voll automatisierten Methoden

R. Stanley

Philosophien und soziale Folgen. Dieser Beitrag soll zeigen, wie sich die Technik in der analytischen Chemie von Schätzungen, Projektionen und manchmal empirischen Resultaten in ein klares Gebiet namens analytische Wissenschaft entwickelte, welches viele Technologien, Strategien und Philosophien umfasste, die früher nicht innerhalb der klassischen Grenzen der Praxis der analytischen Chemie enthalten waren. Obwohl die Geschichte der analytischen Chemie komplex und detailliert ist und das Lebenswerk vieler berühmter Wissenschaftler involviert, scheinen ganz bestimmte Begebenheiten zu existieren, welche die Art und Weise diktierten wie analytische Prozeduren ausgeführt wurden—speziell in der neueren Vergangenheit. Diese Wege öffneten ein weites Gebiet von sozialen und wirtschaftlichen Problemen und Resultaten, welche die Welt des Mikrocomputers lösen oder rechtfertigen will. Deshalb sind analytische Strategien interdisziplinär und multi-dimensional geworden. England scheint es verpasst zu haben das Instrumentengeschäft von heute voll auszuschöpfen. Dazu werden einige Gründe angeführt.

---

### Page 14

### Program in BASIC to combine data from two different selective detectors and its application for screening of pesticides in residue analysis

H.-J. Stan and H. Goebel

The BASIC program presented was for automatic multi-residue analysis of pesticides in food samples. It was designed for the HP 5880 A gas chromatograph, which has dual-channel operation and an autosampler.

The complete procedure: automated sampling, data collection, storage on a cartridge tape, comparison of the quantitative data of each channel and the final analytical report, is controlled via a BASIC program, which makes use of built-in software for peak recognition, integration and calibration.

The capillary column is split to the two selective detectors: ECD and NPD. Data recorded by the two detectors are processed simultaneously and the chromatograms are reported in parallel on two printer/plotters.

The data from the chromatogram are initially stored on tape. Having finished all chromatograms, the data are fed-back into the RAM of the CPU and successively calculated with the various calibration mixtures. The evaluation of all results leads to a final proposal of pesticide residues suspected to be present in the samples.

The program has proved its worth for screening in daily routine analyses for several months.

### Un programme en BASIC pour l'exploitation parallèle des signaux de deux détecteurs de sélectivité différente et son application pour l'analyse des résidus pour le contrôle des pesticides

H.-J. Stan and H. Goebel

Le programme BASIC présenté ici a été développé pour le contrôle automatique de résidus de plusieurs pesticides dans les aliments. Il permet de travailler avec les chromatographes en phase gazeuse HP 5880 A avec deux canaux et échantillonnage automatique. Le programme BASIC contrôle: l'échantillonnage automatique, le traitement des signaux, la mémorisation sur bande magnétique, la comparaison des données quantitatives des deux canaux et le compte-rendu final de l'analyse.

La colonne capillaire mène aux deux détecteurs sélectifs ECD et NPD, et les données des deux détecteurs sont analysées en parallèle. Les chromatogrammes sont présentés simultanément sur deux enregistreurs. Les données bruttes des chromatogrammes sont d'abord mémorisées sur bande magnétique. Après terminaison de tous les chromatogrammes les données sont retransférées dans la mémoire de l'instrument (RAM du CPU) et ensuite comparées avec les différents mélanges de calibrage standard. L'analyse de tous les résultats permet de définir quels sont les résidus de pesticides retrouvés dans les échantillons. Le programme a fait ses preuves dans le contrôle de routine journalier.

**BASIC-Programm für die gleichzeitige Auswertung der Signale von zwei unterschiedlich selektiven Detektoren und seine Anwendung für das Screening von Rückständen in der Pestizidanalytik**
H.-J. Stan and H. Goebel

Das hier dargestellte BASIC-Programm wurde für automatische Vielkomponenten-Pestizidrückstandsanalysen von Lebensmitteln entworfen und für den Gaschromatographen HP 5880 A mit Zweikanalbetrieb und automatischem Probengeber ausgearbeitet.

Über das BASIC-Programm werden gesteuert: Die automatische Probenaufgabe, die Signalverarbeitung, die Speicherung auf ein Magnetband, der Vergleich der quantitativen Daten der beiden Kanäle und der Endreport der Analyse.

Die Kapillarsäule wurde zu den beiden selektiven Detektoren ECD und NPD gesplittet, und die Daten beider Detektoren werden gleichzeitig verarbeitet. Die Chromatogramme werden parallel auf zwei Schreibereinheiten ausgegeben. Die Rohdaten der Chromatogramme werden zuerst auf dem Magnetband gespeichert. Nach Beendigung aller Chromatogramme werden die Daten in den Gerätespeicher (RAM der CPU) zurückgelesen und nacheinander mit den verschiedenen Standard-Eichgemischen verglichen. Die Bewertung aller Meßergebnisse führt zu einem Vorschlag, welche Pestizidrückstände in den Proben vorhanden sein können.

Das Programm hat sich bei der Durchführung des Screenings von täglichen Routineanalysen bewährt.

---

### Page 21

**An evaluation of the Mitsubishi GL-101 glucose analyser**
S. Oldfield *et al.*

An evaluation of the Mitsubishi GL-101 for the measurement of plasma glucose has shown that results compared favourably with those obtained on the Beckman glucose analyser. Although some problems were encountered, the instrument was simple to operate, rapid, and suited to a heavy work-load of routine specimens. Response was linear over a wide range of glucose concentrations and precision was good at high, medium and low glucose concentrations. Several drugs and preservatives were tested and found not to interfere, but haemolysis should be avoided.

**Evaluation du Mitsubishi GL-101 analyseur de glucose**
S. Oldfield *et al.*

L'evaluation du Mitsubishi GL-101 pour la mesure de la glucose dans le plasma a montré que les résultats se comparaient favorablement avec ceux obtenus par un analyseur de glucose Beckman. Bien que certains problèmes ont été rencontrés, l'instrument était facile à opérer, rapide et permet d'analyser un grand nombre d'échantillons de routine. La réponse était linéraire sur un grand domaine de concentrations de glucose et la précision était bonne pour les concentrations élevées de glucose et moyenne pour les concentrations basses. Différents médicaments et préservatifs ont été testés et n'ont montré aucune interférance, mais l'hémolyse doit être évitée.

**Evaluation des Mitsubishi GL-101 Glukose-Analysengeräts**
S. Oldfield *et al.*

Eine Evaluation des Mitsubishi GL-101 für die Bestimmung von Plasma-Glukose hat ergeben, das sich die Resultate gut vergleichen mit denjenigen, die mit dem Beckman Glukose-Analysengerät erhalten werden. Obwohl einige Probleme aufgetreten sind, war das Gerät einfach zu bedienen, schnell und geeignet, eine grosse Arbeitslast von Routineproben zu verarbeiten. Die Messresultate waren über einen weiten Bereich von Glukosekonzentrationen linear, und die Präzision war bei hohen, mittleren und niederen Glukosekonzentrationen gut. Verschiedene Pharmaka und Präservativstoffe wurden geprüft und zeigten keine Interferenzen, aber Hämolyse sollte vermieden werden.

---

### Page 27

**DISNET: a Distributed Instrument System NETwork***
P. J. Gemperline *et al.*

The Distributed Instrument System NETwork (DISNET) is a local area computer network designed to meet the unique needs of laboratory instrumentation. These include high-speed asynchronous communication between micro- and minicomputers, remote control of 'dumb' instruments for real-time data acquisition, and interfacing of expensive peripherals such as plotters and printers to the network for shared access by all network stations. The hardware features byte parallel data transmission and good electrical noise immunity. The salient design features include hardware arbitrated priority access to a party line bus, automatic address recognition, and the ability to reverse the flow of data at any time. In addition, any station in the network may function as a control unit so that no central network master is required. The hardware design and operation is described and initial test results are given. Subsequent work has developed a flexible but simple communication protocol used to interface computer systems to the network.

*French and German abstracts will be published in Vol. 6, No. 2.

---

### Page 33

**An assessment of the IQAS discrete analyser for use in a routine water quality laboratory**
S. Wilson and A. Green

The IQAS discrete analyser and its mode of operation is described. The performance of several water chemistries were optimized and evaluated. Within-batch precisions were found to vary from 0·2 to 0·7, depending on the test. The total error, both within and between batch, varied from 0·7 to 1·7. Carry-over was found to be negligible. Detection limits were found to be an order of magnitude better than autoanalyser systems at present in use at the authors' laboratory.

Comparisons between IQAS and autoanalyser results showed no significant differences except for phosphate where the autoanalyser proved to be at fault.

The IQAS proved in practice to be a much more efficient and accurate way of doing routine analysis in a water laboratory.

**L'évaluation de l'analyseur discret IQAS pour le contrôle chimique de routine de l'eau**
S. Wilson and A. Green

L'analyseur discret IQAS et son mode d'opération sont décrits. La performance de différentes analyses chimiques de l'eau a été optimisée et evaluée. La précision variait de 0,2 à 0,7 dépendant du test. L'erreur total, c'est-à-dire la précision ainsi que l'erreur entre deux séries variaient de 0,7 à 1,7. La contamination était négligeable. Les limites de détection trouvées étaient meilleures d'un ordre de magnitude que pour les systèmes auto-analyseurs utilisés jusqu'à présent dans ce laboratoire. Les comparaisons entre le

IQAS et l'autoanalyseur ne montraient pas de différence significative excepté pour le phosphate où l'auto-analyseur donne des résultats faux. Le travail pratique avec le IQAS a montré que celui-ci est un instrument plus efficace et exact pour l'analyse de routine dans le laboratoire d'eau.

### Beurteilung des diskontinuierlichen IQAS Analysengerätes für Routine-Wasseruntersuchungen im Laboratorium

S. Wilson and A. Green

Die Arbeit beschreibt das diskontinuierliche IQAS Analysengerät und seine Betriebsweise. Die Bestimmung verschiedener Wasseruntersuchungen wurden optimiert und evaluiert. Die Präzision innerhalb einer Serie variierte je nach Test zwischen 0,2 und 0,7. Der Gesamtfehler, innerhalb einer Serie und von Serie zu Serie, variierte zwischen 0,7 und 1,7. Die Probenverschleppung ergab sich als vernachlässigbar. Es zeigte sich, das die Detektionsgrenzen eine Grössenordnung besser sind als bei Autoanalyser-Systemen, die zur Zeit in diesem Laboratorium in Gerbrauch sind.

Ein Vergleich zwischen den IQAS und den Autoanalyser-Resultaten zeigte keine signifikanten Unterschiede mit Ausnahme für Phosphat, wo der Autoanalyser falsche Werte ergab.

Der IQAS zeigte sich in der Praxis als ein sehr viel effizienteres und genaueres Gerät für die Routineanalyse im Wasserlaboratorium.

---

### Page 39

### An evaluation of the Hitachi 705 analyser

C. Ferré *et al.*

The Hitachi 705 automatic analyser was evaluated according to the standard procedure recommended by the Expert Panel on Instrumentation of the 'Sociedad Española de Química Clínica'. The parameters investigated for the evaluation were: within-run and inter-run imprecision, using three different concentration levels; linearity; and contamination effects. The serum constituents analysed were alpha-amylase, creatin kinase, creatin kinase isoenzyme MB, alanine aminotransferase, aspartate aminotransferase, glucose, urea, creatinine, calcium, and protein. In addition, relative accuracy was studied using the SMAC (Technicon Instrument Corporation, Tarrytown, New York, USA) and the ABA-100 (Abbott Laboratories, South Pasadena, California, USA) analysers as comparative methods.

The Hitachi 705 has proved to be a highly reliable and precise instrument. The linearity of the methodology is sufficiently extensive to cover levels of pathological interest without the need for sample dilution.

In the comparative study only a good relative accuracy for calcium and protein (SMAC) and alpha-amylase (ABA-100) was revealed.

### Evaluation de l'analyseur Hitachi 705

C. Ferré *et al.*

L'analyseur automatique Hitachi 705 a été évalué selon les procédures standard recommandées par les experts d'instrumentation de la 'Sociedad Española de Química Clínica'. Les paramètres étudiés pour l'évalutation étaient: précision en série ou entre séries, utilisant trois nivaux de concentration, la linéarité et les effets de contamination. Les constituants de sérum analysé étaient: l'alpha-amylase, la créatine-kinase, l'isoenzyme MB créatine kinase, l'alanine aminotransférase, l'aspartate aminotransférase, la glucose, l'urée, la créatinine, le calcium et les protéines. En plus, l'exactitude relative a été étudiée utilisant le SMAC et le ABA-100 comme instrument de comparaison.

Le Hitachi 705 a fait ses preuves comme instrument de haute précision. La linéarité de la méthode est suffisante pour couvrir des nivaux d'intérêts pathologiques sans la nécessité de diluer les échantillons. Dans l'étude de comparaison uniquement la bonne exactitide relative pour le calcium et le protéine (SMAC) et l'alpha-amylase (ABA-100) ont été étudiés.

### Evaluation des Hitachi 705 Analysenautomaten

C. Ferré *et al.*

Der Hitachi 705 Analysenautomat wurde gemäss dem Standardvorgehen das von der Instrumentierungs-Expertenkommission der 'Sociedad Española de Química Clínica' empfohlen wird evaluiert. Die in der Evaluation untersuchten Parameter waren: Genauigkeit innerhalb und zwischen Serien bei drei verschiedenen Konzentrationen, Linearität, und Probenverschleppungseffekte. Die analysierten Serumbestandteile waren Alpha-Amylase, Kreatin Kinase, Kreatin Kinase Isoenzyme MB, Alanin Aminotransferase, Aspartate Aminotransferase, Glukose, Harnstoff, Kreatinin, Kalzium, und Protein. Zusätzlich wurde die relative Genauigkeit studiert, wobei als Vergleichsmethoden der SMAC und ABA-100 herangezogen wurden.

Der Hitachi 705 erwies sich als ein hochzuverlässiges und präzises Instrument. Die Linearität der Methodologie erstreckt sich über einen genügenden Bereich, um die Bedürfnisse der Pathalogie ohne die Notwendigkeit einer Probenverdünnung abzudecken.

In der Vergleichsstudie wurde eine gute relative Genauigkeit nur für Kalzium und Protein (SMAC) und Alpha-Amylase (ABA-100) gefunden.

---

### Page 44

### Microprocessors in pathology

F. Murphy

This is one of the papers read at the meeting on the Application of Microprocessors in Hospital Practice held on 25 May 1983 by the UK Liaison Committee for Sciences Allied to Medicine & Biology, London.

The author presents the role of the microprocessor, in distinction from the mini or mainframe computer, in pathology. The main areas of application are described and personal experiences of microprocessor implementation in the author's laboratory. These include the BBC microcomputer interfaced to the Gilford 203 System Clinical Analyser and to the Technicon SMA II.

---

### Un analyseur potentiométrique basé sur le micro-ordinateur ZX81

S. W. Bateson *et al.*

Un micro-ordinateur ZX81 a été combiné avec un système d'interface à haute précision pour contrôler un assemblage de titrage potentiométrique. Ceci a été programmé pour une variété d'options incluant le calibrage de la réponse des électrodes. Des résultats illustratifs sont donnés pour le titrage à précipitation de l'argent par le bromure de potassium.

Ein ZX81 Mikrocomputer wurde mit einem Hochpräzisions-Interfacesystem zur Steuerung einer potentiometrischen Titrationsanlage kombiniert. Der Rechner wurde für eine Reihe von Optionen programmiert, inklusive die Herleitung der Eichung des Elektrodenverhaltens. Als erläuterndes Beispiel werden die Resultate für die Fällungstitration von Silbernitrat mit Kaliumbromid angeführt.*

* French and German summaries for 'A potentiometric analyser based on the ZX81 microcomputer' which was published in the last issue of the journal: Vol. 5, No. 4.

